# Meta-analysis and systematic review of intravascular ultrasound versus angiography-guided drug eluting stent implantation in left main coronary disease in 4592 patients

**DOI:** 10.1186/s12872-018-0843-z

**Published:** 2018-06-14

**Authors:** Yue Wang, Gary S. Mintz, Zhichun Gu, Yue Qi, Yue Wang, Mengru Liu, Xiaofan Wu

**Affiliations:** 1 Department of Cardiology, Beijing Anzhen Hospital, Capital Medical University, Beijing, 100029 China; 2 Cardiovascular Research Foundation, New York, NY USA; 3 Department of Pharmacy, Renji Hospital, School of Medicine, Shanghai Jiao Tong University, Shanghai, 200127 China; 4 Department of Epidemiology, The Key Laboratory of Remodeling-Related Cardiovascular Diseases, Ministry of Education, Beijing Institute of Heart, Lung and Blood Vessel Diseases, Beijing Anzhen Hospital, Capital Medical University, Beijing, China

**Keywords:** Intravascular ultrasound, Angiography, Drug eluting stent, Left main disease, Meta-analysis

## Abstract

**Background:**

Although several meta-analyses have demonstrated the utility of intravascular ultrasound (IVUS) in guiding drug-eluting stent (DES) implantation compared to angiography-guidance, there has been a dearth of evidence in the left main coronary artery (LMCA) lesion subset.

**Methods:**

We performed a meta-analysis to compare clinical outcomes of IVUS versus conventional angiography guidance during implantation of DES for patients with LMCA disease. Pubmed, Cochrane Library, Embase were searched.

**Results:**

A total of 1002 publications were reviewed; and finally, seven clinical studies - one prospective randomized controlled trial and six observational studies with 4592 patients (1907 IVUS-guided and 2685 angiography-guided) - were included in the meta-analysis. IVUS guidance was associated with a significant reduction in major adverse cardiac events (relative ratio [RR] 95% CI 0.61; 95% confidence interval [CI] 0.53 to 0.70; *P* < 0.001), all-cause death (RR 0.55; 95% CI 0.42 to 0.71; *P* < 0.001), cardiac death (RR 0.45; 95% CI 0.32 to 0.62; *P* < 0.001), myocardial infarction (RR 0.66; 95% CI 0.55 to 0.80; *P* < 0.001), and stent thrombosis (RR 0.48; 95% CI 0.27 to 0.84; *P* = 0.01) compared with angiographic guidance. However, there was no significant difference regarding target lesion revascularization (RR 0.60; 95% CI 0.31 to 1.18; *P* = 0.099) and target vessel revascularization (RR 0.64; 95% CI 0.26 to 1.56; *P* = 0. 322).

**Conclusions:**

Compared to angiographic guidance, IVUS-guided DES implantation was associated with better clinical outcomes for patients with LMCA lesions, especially hard endpoints of death, myocardial infarction, and stent thrombosis.

## Background

Intravascular ultrasound (IVUS) has played a key role in contemporary stent-based percutaneous coronary interventions (PCI) by providing more detailed coronary anatomic information, assessing plaque burden accurately, selecting proper stent sizes, and optimizing stent expansion, apposition, and geographic miss [1–5]. One meta-analysis of seven randomized trials in the bare metal stent era [6] and seven meta-analyses including both registries and randomized studies [7–13] in the drug-eluting stent (DES) era concluded that IVUS guidance improved patient outcomes compared to angiography guidance alone. However, and with one exception, these previously published meta-analyses did not address stent implantation to treat the subset of patients who present for PCI of left main coronary artery (LMCA) lesions; and there is increased interest in PCI intervention for LMCA disease since the results of two randomized DES versus bypass surgery studies – EXCEL and NOBLE – were recently presented [14, 15]. Therefore, we performed the current meta-analysis of published studies comparing IVUS-guided versus angiography-guided DES implantation to treat LMCA lesions.

## Methods

### Data sources and search strategy

A computerized search was performed of Pubmed, Cochrane Library, Embase from January 1993 to October 2017. Mesh and combinations of the following terms were used in the search process: “ultrasonography, intravascular,” “intravascular ultrasound,” “intravascular ultrasound-guided,” “IVUS,” “IVUS-guided,” “angiography,” “angiography-guided,” “left main coronary artery,” “left main coronary stenosis,” “left main coronary disease,” “left main,” “left main lesion,” “LMCA,” “drug-eluting stent,” “sirolimus-eluting stent,” “paclitaxel-eluting stent,” “everolimus-eluting stent,” “zotarolimus-eluting stent,” “stent,” and “DES.” Two investigators (Yue Wang^*^ and Yue Qi) independently screened the titles and abstracts and eventually examined the full texts of the original reports included in the study. Additional searches for potential studies were performed by reviewing the references of earlier meta-analyses concerning IVUS versus angiography-guided DES implantation. Complete data were retrieved from the studies for quantitative synthesis. In addition, studies with incomplete information - including abstracts of major meetings (Transcatheter Cardiovascular Therapeutics [TCT], Angioplasty Summit, American Heart Association [AHA], American College of Cardiology [ACC], EuroPCR, and World Congress of Cardiology [WCC]) and studies of IVUS versus angiography-guided DES implantation that included a *subgroup* of LMCA patients - were reviewed for other potentially relevant citations.

### Selection criteria

Final inclusion of studies was based on the agreement of both reviewers. Randomized control trials (RCTs) and observational studies in English language were considered while studies in the non-English language literature were not included. Studies met the following pre-specified criteria: 1) clinical studies published in peer-reviewed journals with fully available data; 2) studies that included a comparison of IVUS-guided versus angiography-guided PCI with DES in LMCA lesions; and 3) follow-up time of at least 12 months. Reports of mixed treatment with bare-metal stent and DES implantation without separate clinical outcomes for the DES subgroup were excluded. Within this study, [16] patients (*n* = 1899) predominantly underwent DES implantation, with BMS implanted in only a small proportion of the study population (IVUS-guided arm: 1.3%, angiography-guided arm: 3.0%). It was therefore deemed appropriate to include this study in the meta-analysis. In addition, studies with incomplete data were reviewed and later discussed, but not included in the formal meta-analysis.

### Endpoints and definition

The primary endpoint in this meta-analysis was major adverse cardiac events (MACE), defined as the composite of death, MI (myocardial Infarction), and repeat revascularization. The secondary endpoint was all-cause death, cardiac death, MI, target lesion revascularization (TLR), target vessel revascularization (TVR) and stent thrombosis (ST; included definite, probable or possible ST) according to the definition of the Academic Research Consortium [17].

### Data extractions

We extracted DES data exclusively, thereby excluding bare metal stent data. The study’s first author’s name, publication date, study design, and follow-up duration; baseline clinical, angiographic, and procedural characteristics; and clinical outcomes were systematically reviewed and recorded by the same two reviewers (Yue Wang^*^ and Yue Qi). Disagreements were resolved by discussion between them.

### Quality assessments

The methodological quality of RCTs was assessed by the Cochrane Collaboration Risk of Bias tool [18]. The methodological quality of observational studies was assessed by the Newcastle-Ottawa scale (NOS) that consists of three factors: patient selection, comparability of the study groups, and assessment of outcomes [19]. A score of 0–9 was allocated to each study except RCTs. Observational studies achieving six or more scores were considered to be of high quality [20].

### Statistical analyses

Baseline characteristics between IVUS-guided versus angiography-guided groups were analyzed and compared, with mean ± SD for continuous variables using two-sample student’s unpaired t-test and proportions for categorical variables using chi-square statistics. Across-study summary relative ratios (RRs) with 95% confidence interval (CI) were produced to assess the efficacy of IVUS versus angiography guidance on adverse clinical events. The statistical heterogeneity between trials was assessed with chi-square tests and I^2^ statistics. When the *p* value of chi-square test was < 0.10 and/or the I^2^ was ≥50%, significant heterogeneity was considered and a random-effects model would be selected. If not, the fixed-effects model was used instead. Egger’s linear regression analysis was performed to quantitatively assess the underlying publication bias across the studies. In order to evaluate the stability and reliability of the primary endpoint result, we performed a sensitivity analysis of MACE by omitting each individual study in turn. All reported *p*-values were 2-sided, and *P* < 0.05 was considered to indicate statistical significance. All statistical analyses were performed using STATA 14.0 (Stata Corp, College Station, TX, USA).

## Results

### Studies included

**Fig. 1:**
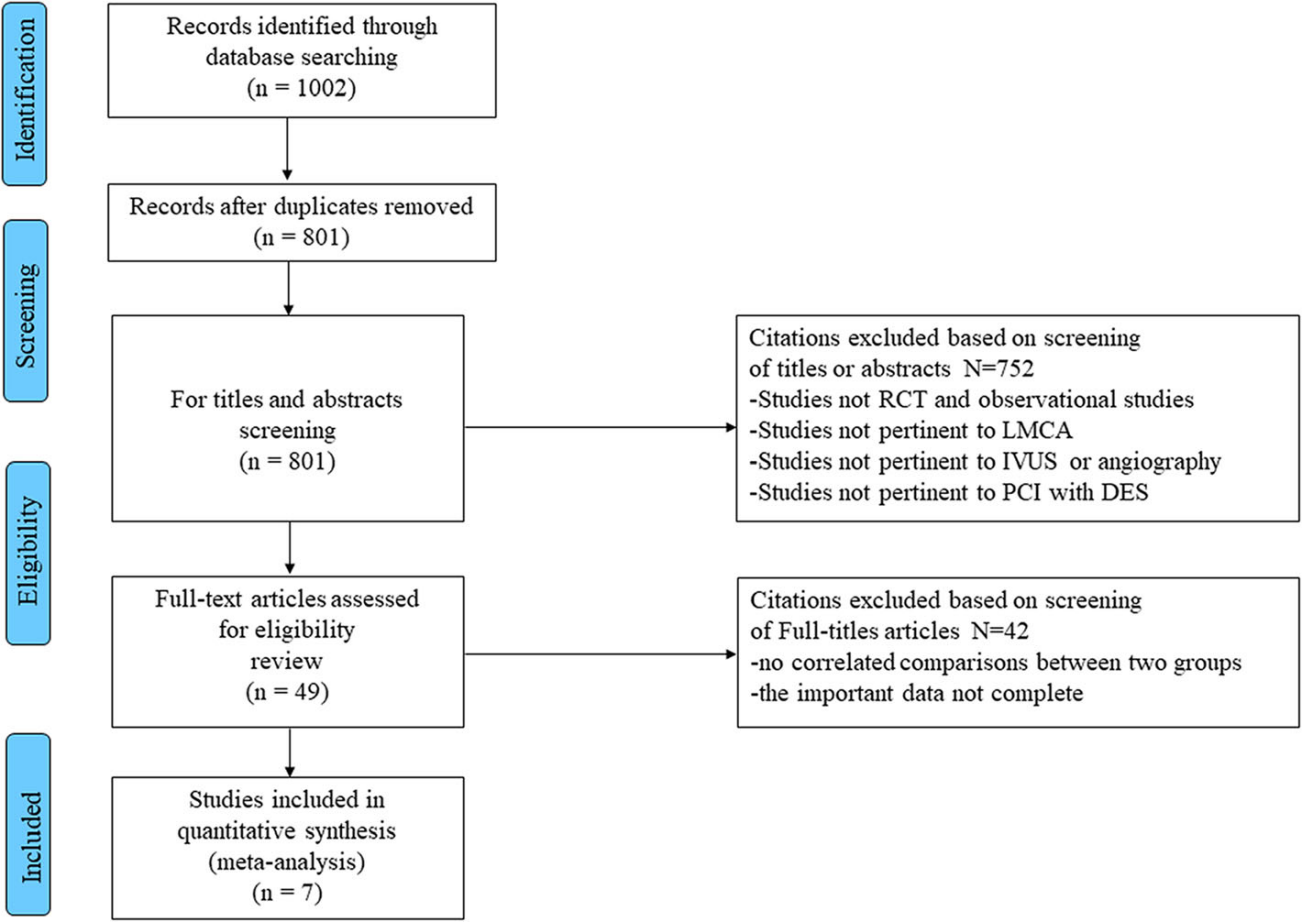
Flow diagram of the process followed to identify the relevant studies that were included in the present meta-analysis

A total of 1002 publications were reviewed, and 801 citations were screened by checking the title or abstract. Of these, 49 studies were reviewed in detail; and seven clinical studies (4592 patients) were included in the current formal meta-analysis, [16, 21–26] in which 1907 patients underwent IVUS-guided PCI and 2685 underwent angiography-guided PCI with DES implantation. (Fig. 1) One study was a RCT, [25] and the other six studies were observational registries [16, 21–24, 26]. Four studies performed propensity score matching [16, 22, 23, 26]. As specified in the Methods, one meeting abstract published in 2016 (Effectiveness and safety of intravascular ultrasound guidance on clinical outcomes following drug-eluting stent implantation in unprotected left main coronary artery) and one study [27] that included LMCA subgroup analysis were reviewed in the Discussion, not included in our meta-analysis.

### Study characteristics

Baseline characteristics of the included studies have been shown in Table 1. The follow-up time ranged from 12 to 36 months. Of the seven studies, the follow-up time in four was 36 months. There were no statistically significant differences in the baseline characteristics between the IVUS-guided versus the angiography-guided groups, except for age, left ventricular ejection fraction and previous PCI. Table 2 demonstrating angiographic and procedural characteristics. Four studies mainly used first generation DES [21, 22, 24, 25], and both first and second-generation DES were implanted in two studies [16, 26]. However, one study did not report the type of DES [23]. Most studies were frequent use of a two-stent technique except for one study by Kim [26].

**Table 1:** Study design, baseline characteristics of the included studies

First author	Year	Design	Sample size	F/U (months)	Age (years)	Male	Smoker	DM	Hyperlipidemia	Hypertension	Renal failure	LVEF %	Previous PCI	Previous MI
Agostoni et al. [21]	2005	Observational Unadjusted	24/34	14	62/64	15/25	4/7	9/10	15/23	14/20	–	52/44^a^	12/17^a^	9/17
Park et al. [22]	2009	Observational Adjusted	145/145	36	64/65^a^	102/102	28/30	49/49	42/44	86/85	110/112	60/61	–	10/11
De La Torre Hernandez et al. [23]	2014	Observational Adjusted	505/505	36	66/67	404/397	148/161	183/175	314/284	342/325	35/31	55/55	111/107	122/130
Gao et al. [24]	2014	Observational Unadjusted	337/679	12	66/67	274/526	111/230	109/232	228/487	244/489	88/214	59/57^a^	60/119	60/123
Tan et al. [25]	2015	RCT	61/62	24	77/76	38/43	27/29	21/18	–	25/29	–	55/53	–	10/13
Kim et al. [26]	2017	Observational Adjusted	122/74	36	62/65^a^	95/53	60/37	47/33	28/17	75/54	10/7^b^	55/49^a^	9/14^a^	4/5
Tian et al. [16]	2017	Observational Adjusted	713/1186	36	60/60	576/920	256/316	173/314	387/597	400/654	–	63.2/62.9	165/270	163/293

Data are presented as IVUS-guided/angiography-guided PCI with DES for left main lesions

*DM* diabetes mellitus, *F/U* follow-up, *LVEF* left ventricular ejection fraction, *MI* myocardial infarction, *PCI* percutaneous coronary intervention, *RCT* randomized controlled trial

^a^*p* < 0.05

^b^Chronic kidney disease

**Table 2:** Angiographic and procedural characteristics

First author	Lesion number	Ostial lesion	Midshaft lesion	Distal lesion	LM only	LM plus 1 Vessel disease	LM plus 2 vessel disease	LM plus 3 vessel disease	Types of Stent	Stent number	Stent diameter (mm)	Stent Length (mm)	Two-stent technique	Final kissing balloon	Post-dilation
Agostoni et al. [21]	–	7/3	7/9	10/22	–	–	11/25	–	SES/PES	1.5/1.4	3.2/3.2	27/23	7/11	4/10	22/26
Park et al. [22]	–	61/62^a^		84/83	6/8	35/32	48/48	56/57	SES/PES	1.23/1.24	–	35.16/35.63	39/42	–	–
De La Torre Hernandez et al. [23]	1.47/1.5	151/145	133/134	221/226	–	160/168	161/149	–	–	–	3.8/3.65	16.0/16.8	63/62	–	–
Gao et al. [24]	1.2/1.3	32/59	16/30	191/359	–	–	–	–	SES	1.5/1.4	3.5/3.4	35.4/33.3	154/280	–	321/543
Tan et al. [25]	–	29/28^a^		32/34	7/10	14/13	24/22	16/17	SES	1.39/1.42	3.43/3.44	21.48/18.24	24/26	–	23/9
Kim et al. [26]	–	–	–	–	–	–	–	–	First and second-generation	–	–	25/28	0/0	26/12	–
Tian et al. [16]	1.7/1.68	81/148	45/82	587/956	59/66	141/240	258/416	255/464	First and second-generation	–	3.54/3.39	21.7/24.3	266/236	399/395	551/638

Data are presented as IVUS-guided /angiography-guided PCI with DES for left main coronary artery lesions

*SES* sirolimus eluting stent, *PES* paclitaxel eluting stent

^a^Ostial or midshaft LM lesions

### Assessment of quality

Table 3 presented quality assessment results of included observational trials. Among the six observation studies, all had a scoring of ≥6. The summary risk of bias of the RCT study was low. All studies were considered to be of high quality.

**Table 3:** Quality assessment results of included observational trials

First author	Year	Selection				Comparability	Outcomes			
		Representativeness of the exposed cohort	Selection of the unexposed cohort	Ascertainment of exposure	No interest outcomes at start of the study	Comparability of cohorts on the basis of design and analysis	Assessment of outcomes	Was follow-up sufficient for the outcome	Adequacy of follow-up	NOS score
Agostoni et al. [21]	2005	*	*	*	*			*	*	6
Park et al. [22]	2009	*	*	*	*	**		*	*	8
De La Torre Hernandez et al. [23]	2014	*	*	*	*	**	*	*		8
Gao et al. [24]	2014	*	*	*	*	**	*	*		6
Kim et al. [26]	2017	*	*	*	*		*	*		6
Tian et al. [16]	2017	*	*	*	*	*	*	*	*	8

Footnote: Each asterisk represents one star in the Newcastle-Ottawa Scale system. The maximum number of stars is 2 for comparability and 1 for each of the other categories, for a total of up to 9 stars. *NOS* Newcastle-Ottawa scale

### Clinical outcomes

**Fig. 2:**
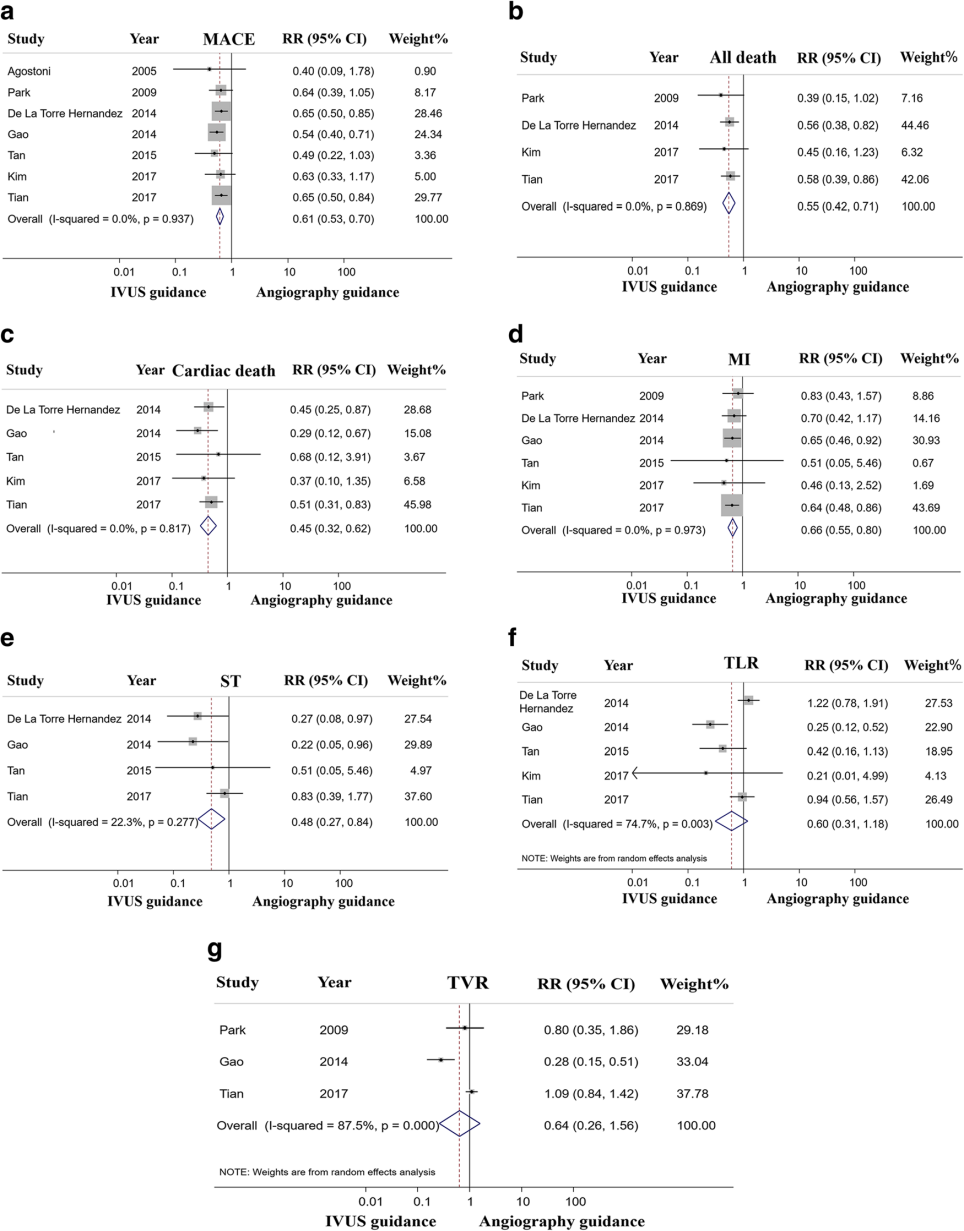
Forest plot of RR for MACE (**a**), all cause death (**b**), cardiac death (**c**), MI (**d**), ST (**e**), TLR (**f**) and TVR (**g**) associated with IVUS guided vs angiography guided DES implantation to treat LMCA disease. Squares is the effect size of the individual studies; diamonds, the summarized effect size; horizontal lines, upper and lower border of 95% confidence interval. DES = drug eluting stent; LMCA = left main coronary artery; IVUS = intravascular ultrasound; MACE = major adverse cardiac event; MI = myocardial infarction; relative ratio = RR; ST = stent thrombosis; TLR = target lesion revascularization; TVR = target vessel revascularization

Analyses for clinical outcomes have been presented in Fig. 2. The definition of MACE was slightly different across studies; two studies included cardiac death, [21, 25] and the other five included all-cause deaths [16, 21–24, 26]. Three studies reported TVR, [21, 22, 24] two reported TLR, [23, 25] and one study reported any revascularization not restricted to the target lesion [26] (Table 4).

**Table 4:** The endpoint events and definitions

First author	MACE	Repeat revascularization	Stent thrombosis
Agostoni et al. [21]	Cardiac death, MI, TVR	–	–
Park et al. 22]	All cause death, MI, TVR	TVR	–
De La Torre Hernandez et al. [23]	All cause death, MI, TLR	TLR	Definite, probable
Gao et al. [24]	All cause death, MI, TVR	TLR, TVR	Definite, probable, possible
Tan et al. [25]	Cardiac death, MI, TLR	TLR	Definite, probable
Kim et al. [26]	All cause death, MI, TVR, TLR	TLR, TVR	–
Tian et al. [16]	All cause death, MI	TLR, TVR	Definite, probable

*MACE* major adverse cardiovascular event, *MI* myocardial infarction, *TLR* target lesion revascularization, *TVR* target vessel revascularization

MACE was reported in all seven studies (Table 5), the summary RR was 0.61 (95% CI 0.53 to 0.70; *P* < 0.001) in favor of IVUS-guided DES implantation. No evidence of statistical heterogeneity was noted among the included studies (*I*^*2*^ = 0%; *P* = 0.937) (Fig. 2a).

**Table 5:** Clinical outcomes for MACE in the IVUS-guided and angiography-guided groups

First author	Year	Sample size	IVUS guidance^a^	Angiography guidance^a^	RR	95% CI	*P*-value
Agostoni et al. [21]	2005	24/34	8%	20%	0.4	0.09–1.78	0.18
Park et al. [22]	2009	145/145	–	–	0.64	0.39–1.05	0.074
De La Torre Hernandez et al. [23]	2014	505/505	14.4%	22.2%	0.65	0.5–0.85	0.006
Gao et al. [24]	2014	337/679	14.8%	27.7%	0.54	0.4–0.71	< 0.001
Tan et al. [25]	2015	61/62	13.1%	27.4%	0.49	0.22–1.03	0.031
Kim et al. [26]	2017	122/74	21%	43%	0.63	0.33–1.17	0.149
Tian et al. [16]	2017	713/1186	5.3%	8.1%	0.65	0.5–0.84	0.001

*CI* confidence interval, *MACE* major adverse cardiac events, *RR* relative ratio

^a^Percentage of total population

Four studies reported all-cause death and indicated that IVUS-guided DES implantation was associated with a significant reduction of all-cause mortality (RR 0.55; 95% CI 0.42 to 0.71; *P* < 0.001) with no statistical heterogeneity across the studies (*I*^*2*^ = 0%; *P* = 0.869) (Fig. 2b).

Of the seven studies, five were included in the analysis of cardiac death; the risk of cardiac death was significantly lower with IVUS guidance (RR 0.45; 95% CI 0.32 to 0.62; *P* < 0.001) with no statistically significant heterogeneity (*I*^*2*^ = 0%; *P* = 0.817) (Fig. 2c).

Six studies were applied to the analysis of MI. The result was significantly in favor of IVUS-guided DES implantation (RR 0.66; 95% CI 0.55 to 0.80; *P* < 0.001) with no heterogeneity (*I*^*2*^ = 0%; *P* = 0.973) (Fig. 2d).

Data on ST was reported in four studies. In PCI with DES implantation, IVUS-guidance markedly lowered the risk of definite/probable ST compared with the angiography guidance group (RR 0.48; 95% CI 0.27 to 0.84; *P* = 0.01), again with no statistical heterogeneity (*I*^*2*^ = 22.3%; *P* = 0.277) (Fig. 2e).

Six studies reported data regarding TLR and three reported TVR. Due to significant heterogeneities (TLR *I*^*2*^ = 74.7%; *P* = 0.003, and TVR *I*^*2*^ = 87.5%; *P* < 0.001), random effects models were used to estimate the summary effect of all studies. There were no significant statistical differences regarding TLR (RR 0.60; 95% CI 0.31 to 1.18; *P* = 0.099) and TVR (RR 0.64; 95% CI 0.26 to 1.56; *P* = 0. 322) between the two groups (Fig. 2f and Fig. 2g).

### Sensitivity analysis

**Fig. 3:**
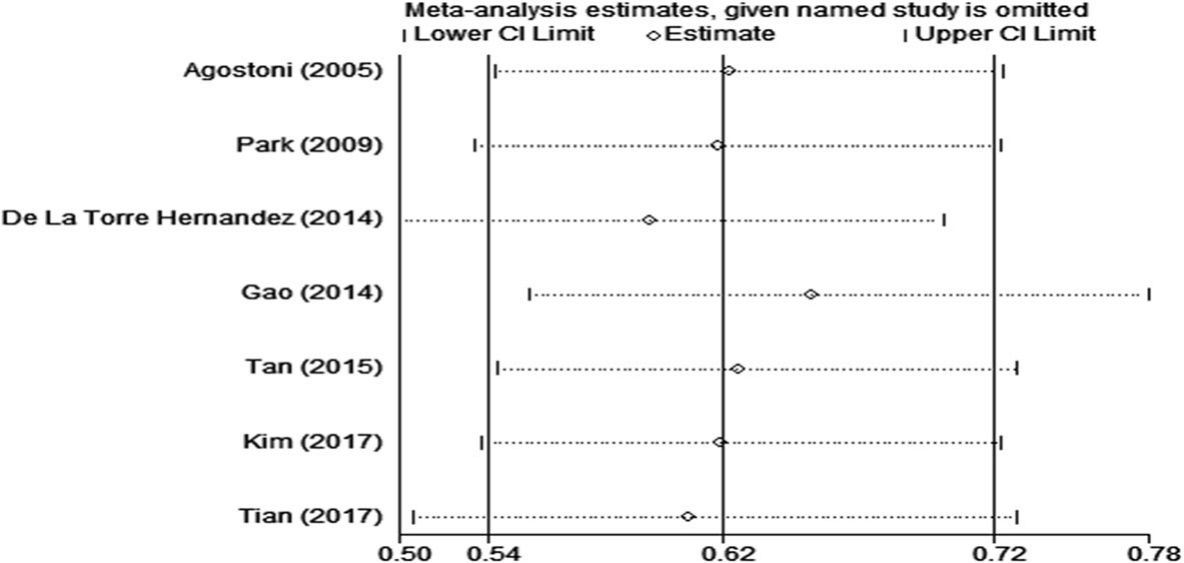
Sensitivity analysis of MACE by omitting each individual study in turn. Cycle is relative ratio; horizontal lines, upper and lower border of 95% confidence interval. MACE = major adverse cardiac event

Sensitivity analysis regarding the primary endpoint MACE has been presented in Fig. 3. After removing each study in sequence, the results were not statistically different from the summary RR across the seven studies, further indicating that IVUS-guided DES implantation was associated with a significant reduction in MACE.

### Publication bias

**Fig. 4:**
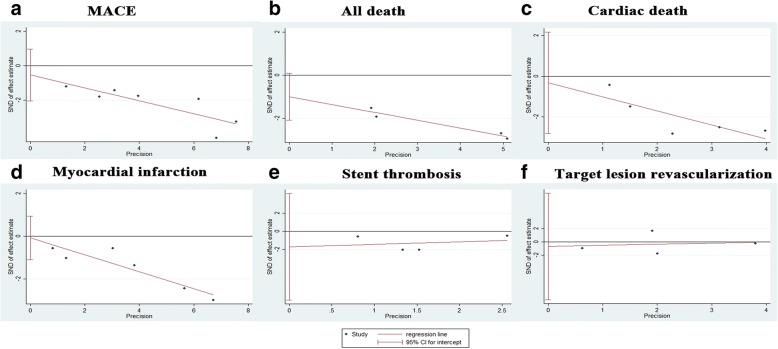
Assessment of publication bias using the Egger’s linear regression analysis for MACE (**a**), all cause death (**b**), cardiac death (**c**), MI (**d**), ST (**e**), TLR (**f**). MACE = major adverse cardiac event; MI = myocardial infarction; ST = stent thrombosis. TLR = target lesion revascularization; TVR = target vessel revascularization

Because only seven clinical studies were included the current meta-analysis, we assessed the asymmetry of publication using quantitative tools. No significant evidence of publication bias (*P* = 0.401 for MACE, *P* = 0.058 for all death, *P* = 0.709 for cardiac death, *P* = 0.842 for MI, *P* = 0.338 for ST and *P* = 0.75 for TLR) were observed on the basis of Egger’s test (Fig. 4).

## Discussion

This meta-analysis of seven studies consisting of 4592 patients demonstrated that IVUS-guided DES implantation when treating LMCA lesions was associated with a significantly reduced risk of MACE, all-cause death, cardiac death, MI, and ST, when compared with angiography-guidance.

Using coronary angiography, LMCA lesions may be obscured by overlapping vessels, streaming of injected contrast, and lack of a normal reference segment that limits the utility of angiography in determining lesion severity and selecting treatment strategy [28]. A study by Chakrabarti demonstrated that 11.2% (17 of 152) patients with LMCA disease by core laboratory assessment were deemed normal by clinical assessment in the National Cardiovascular Data Registry (NCDR), whereas 56.7% (177 of 312) patients having significant LMCA disease by clinical assessment in the NCDR had no LMCA lesions by core laboratory analysis [29]. In addition, visual inspection of the angiogram may result in an incorrect diagnosis of LMCA disease severity. In one study including 213 patients with LMCA stenosis, 23% of patients had a diameter stenosis < 50% on the angiogram while the FFR was < 0.80, who actually had hemodynamically significant stenosis and needed revascularization [30].

Conversely, IVUS can detect significant narrowing and assess angiographically ambiguous LMCA lesions; [31] and IVUS correlates with FFR [32]. IVUS has been used to guide decision-making with regards to PCI or bypass surgery in patients with LMCA disease [33]. IVUS also has the ability to assess LMCA plaque distribution and predict hemodynamically significant jailing of the left circumflex after single stent cross-over in a manner not possible using coronary angiography [34, 35]. More importantly, IVUS can assess stent underexpansion, incomplete lesion coverage (ie, large residual stent edge plaque burden), and malapposition in both LMCA and non-LMCA lesions after DES implantation [36, 37]. Optimal DES implantation is the key to improved patient outcomes. A multivariable logistic regression model of PCI treatment of LMCA lesions showed that IVUS-determined stent underexpansion was an independent predictor for MACE (adjusted hazard ratio [HR] = 5.56; *P* < 0.001); and the two-year MACE-free survival rate was significantly lower in patients with underexpansion of at least one segment versus lesions with no underexpansion (90% vs 98%; *P* < 0.001) [37].

The current meta-analysis demonstrating that IVUS-guided DES implantation led to a significant reduction in the incidence of MACE compared with angiography guidance. This has also been seen in several abstracts at major meetings, and in LMCA subset analyses of larger IVUS versus angiography-guided DES implantation studies. For example, a recently published summary in WCC_2016 concluded that the risk of MACE was significantly lower in LMCA lesions stented with IVUS guidance (15%) vs angiography guidance (24%) (X^2^ = 42.76; *P* = 0.009). In addition, the subset analyses of LMCA patients in the ADAPT-DES study also showed a trend toward a reduction in MACE (HR 0.54; 95% CI 0.23–1.26; *P* = 0.15) [30].

The beneficial results of IVUS-guidance demonstrated in the current meta-analysis were mainly related to the lower risk of death and MI. In addition, the use of IVUS was associated with a lower ST risk than reliance on angiography. Considering that ST within LMCA stents may present as sudden death, the incidence of this complication may have been underestimated, but was likely captured in the assessment of patient mortality and/or MI [22].

However, we found that IVUS guidance did not reduce TLR or TVR. This may be attributed to the fact that underpowered study population, low incidence of events and the discretion of the operator who might prefer IVUS guidance for lesions with more complex coronary anatomy.

### Limitations

This meta-analysis had the following limitations. Firstly, only seven studies were included in our meta-analysis; and only one was an (admittedly relatively small) RCT. Indeed, observational studies have significant limitations with selection and ascertainment bias, which influenced the quality of the evidence across studies. In the present meta-analysis, four of six observational studies included propensity score matching to reduce selection biases. Moreover, IVUS-guided DES implantation was still associated with a significant reduction in MACE after sensitivity analysis was performed. The result indicated that IVUS was worthy of being recommended while performing DES placement for LMCA. However, adequately powered, large-scale RCT studies are needed. Secondly, the location of LMCA lesions, the numbers of other diseased vessels, DES type, and specific treatment strategies may also have impacted the clinical outcomes; but most studies did not provide this detailed data so that subgroup analyses could not be conducted. Thirdly, this meta-analysis lacked the power to detect meaningful differences in TLR and TVR due to little sample size and significant heterogeneities.

## Conclusions

The current meta-analysis and systematic review of the literature demonstrate potential clinical value of IVUS in guiding DES implantation for LMCA patients by a significantly reduced risk of MACE, all-cause death, cardiac death, MI, and ST compared with angiography guidance alone. Future studies are warranted as more data becoming available.

## Abbreviations

ACC: American College of Cardiology
AHA: Angioplasty Summit, American Heart Association
CI: Confidence interval
DES: Drug eluting stent
FFR: Fractional flow reserve
HR: Hazard ratio
IVUS: Intravascular ultrasound
MACE: Major adverse cardiac event
MI: Myocardial infarction
NCDR: National Cardiovascular Data Registry
NOS: Newcastle-Ottawa scale
PCI: Percutaneous coronary interventions
PES: Paclitaxel eluting stent
RCTs: Randomized control trials
RRs: Relative ratios
SES: Sirolimus eluting stent
ST: Stent thrombosis
TCT: Transcatheter Cardiovascular Therapeutics
TLR: Target lesion revascularization
TVR: Target vessel revascularization
WCC: World Congress of Cardiology
